# Noninvasive genotyping and monitoring of anaplastic lymphoma kinase (ALK) rearranged non-small cell lung cancer by capture-based next-generation sequencing

**DOI:** 10.18632/oncotarget.11569

**Published:** 2016-08-24

**Authors:** Ye Wang, Pan-Wen Tian, Wei-Ya Wang, Ke Wang, Zhou Zhang, Bo-Jiang Chen, Yan-Qi He, Lei Li, Hao Liu, Shannon Chuai, Wei-Min Li

**Affiliations:** ^1^ Department of Respiratory and Critical Care Medicine, West China Hospital, Sichuan University, Chengdu, Sichuan Province, China; ^2^ Lung Cancer Treatment Center, West China Hospital, Sichuan University, Chengdu, Sichuan Province, China; ^3^ Department of Pathology, West China Hospital, Sichuan University, Chengdu, Sichuan Province, China; ^4^ Burning Rock Biotech, Guangzhou, Guangdong Province, China

**Keywords:** capture-based next-generation sequencing, ALK fusion, ALK rearrangements, cell-free DNA, non-small cell lung cancer

## Abstract

Noninvasive genotyping of driver genes and monitoring of tumor dynamics help make better personalized therapeutic decisions. However, neither PCR-based assays nor amplicon-based targeted sequencing can detect fusion genes like anaplastic lymphoma kinase (ALK) rearrangements in blood samples. To investigate the feasibility and performance of capture-based sequencing on *ALK* fusion detection, we developed a capture-based targeted sequencing panel to detect and quantify rearrangement events, along with other driver mutation variants in plasma. In this perspective study, we screened 364 patients with advanced non-small cell lung cancer (NSCLC) for *ALK* rearrangements, and collected blood samples from 24 of them with confirmed *ALK* rearrangements based on their tissue biopsies. ALK rearrangements were successfully detected in 19 of 24 patients at baseline with 79.2% (95% CI 57.9%, 92.9%) sensitivity and 100% (36/36) specificity. Among the 24 patients, we obtained longitudinal blood samples from 7 of them after either chemotherapy and/or Crizotinib treatment for disease monitoring. The by-sample detection rate of ALK rearrangements after treatment drops to 69.2% (9 of 13). In addition to detecting ALK rearrangements, we also detected 3 Crizotinib resistant mutations, ALK L1152R, ALK I1171T and ALK L1196M from patient P4. ctDNA concentration correlates with responses and disease progression, reflecting its ability as a biomarker. Our findings suggest capture-based sequencing can detect and quantify *ALK* rearrangements as well as other somatic mutations, including mutations mediated drug resistance, in plasma with high sensitivity, paving the way for its application in identifying driver fusion genes and monitoring tumor dynamics in the clinic.

## INTRODUCTION

Small-molecule tyrosine kinase inhibitors (TKIs) targeting driver oncogenic mutations have been developed to treat advanced NSCLC and have brought significant clinical benefits to patients [1–4]. Crizotinib and other ALK inhibitors are efficacious to treat patients with *ALK* rearrangements, which account for 2–7% of all NSCLC [4, 5]. Current *ALK* rearrangements assays include fluorescence *in situ* hybridization (FISH), RT-PCR and immunohistochemistry (IHC), which are usually conducted on tissue biopsies before commencing treatments [6, 7]. However, tissue samples are sometimes insufficient for a comprehensive diagnosis or gene profiling. Under some circumstances, biopsies are inaccessible, particularly at time of disease progression. Furthermore, FISH results are often subjective, and tumor cells harboring *ALK* rearrangements may distribute asymmetrically due to intra-tumor heterogeneity [8]. These limitations for tissue-based *ALK* rearrangements assays highlight the potential benefits of an alternative noninvasive means to identify NSCLC patients with *ALK* rearrangements.

There have been major advancements in noninvasive detection and assessment of EGFR driver mutations [9–13]. In addition to high detection sensitivity, quantification of ctDNA by droplet digital PCR (ddPCR) makes it possible to monitor tumor burden noninvasively, and trace resistant T790M mutation before clinical progression of disease [13, 14]. However, it is difficult to detect rearrangement events such as *ALK* and *ROS1* in ctDNA with PCR-based assays, because the rearrangement breakpoints reside on broad intronic regions without apparent “hot spots”, making it difficult to design PCR primers. Capture-based targeted deep sequencing provides an economical and ultrasensitive method for detecting mutations, copy number variations, and rearrangement events simultaneously on genomic regions of interested for up to a few million base pairs. Using such technique, *EML4-ALK* fusion and two previously unreported fusions involving *ROS1* were identified from plasma DNA in an advanced NSCLC cohort [15]. Another study reported a 75% sensitivity of rearrangements detection from cfDNA of 8 patients, while 2 out of the 3 *ALK* rearrangements were successfully identified [16]. In the meanwhile, capture-based targeted sequencing can also quantify the allelic fraction of genetic variants, and has therefore become highly useful for tumor burden surveillance and for elucidating possible treatment resistance mechanisms [17]. Deep sequencing of plasma DNA has been applied on EGFR mutation detection and the sensitivity was reported to be 72.7% in advanced NSCLC [18]. However, very few studies have reported such type of clinical application for *ALK* rearrangements. In this study, we assessed the feasibility and performance of applying targeted deep sequencing to plasma cfDNA to detect and quantify *ALK* rearrangements as well as other somatic mutations to survey tumor dynamics.

## RESULTS

### Patient characteristics

**Figure 1:**
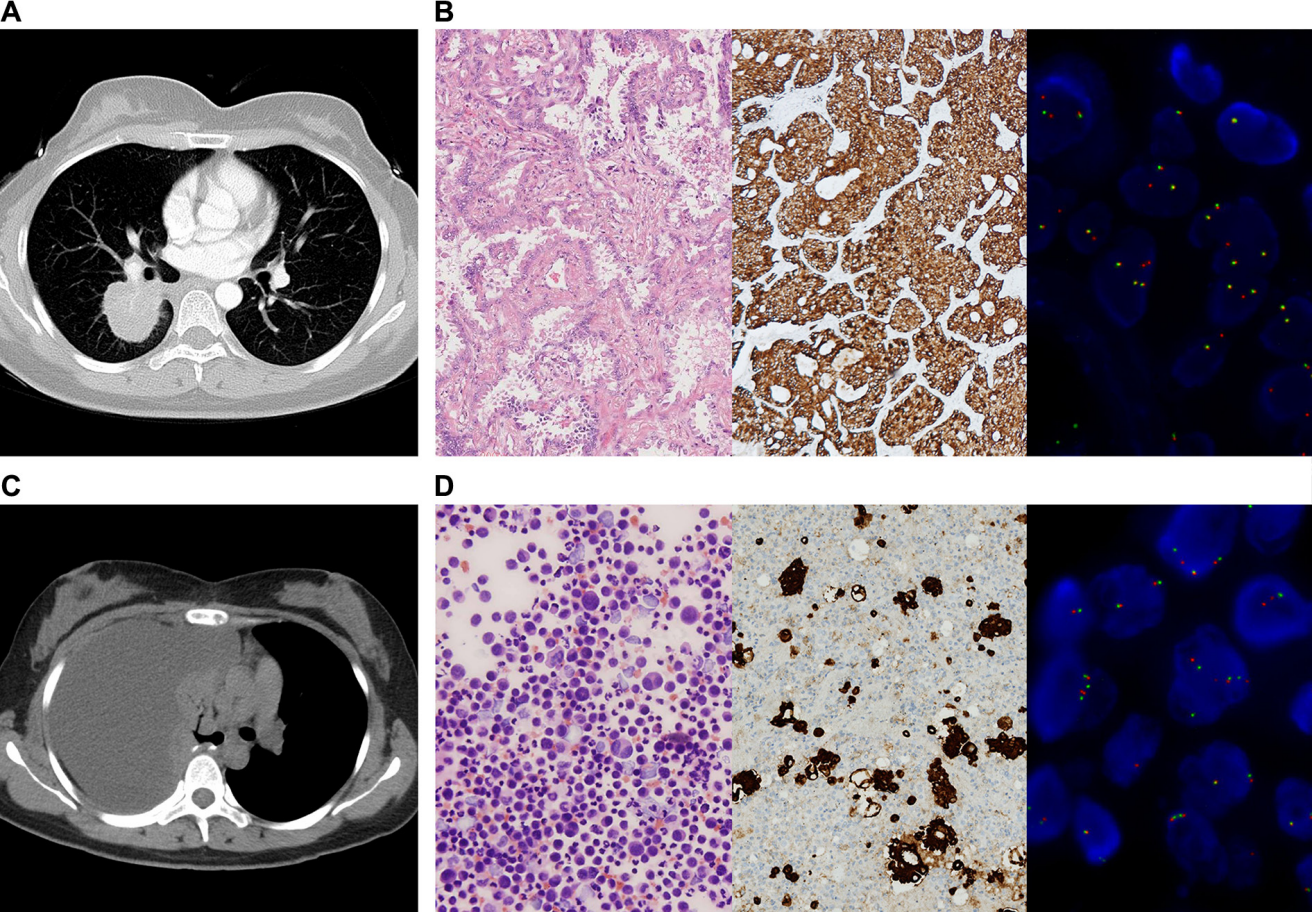
IHC and FISH confirmed the presence of ALK rearrangements in tumor tissue and cell block (**A**) CT scan showed a mass in the right lower lobe of a 31-year-old female (P1). She completed an adjuvant chemotherapy after lobectomy. (**B**) HE staining of her tumor tissue revealed adenocarcinoma, and *ALK* rearrangement was confirmed by IHC (Ventanna) and FISH. (**C**) Three months after adjuvant chemotherapy, CT scan showed massive right-side pleural effusion. (**D**) Adenocarcinoma cells were found in cell block of pleural effusion. IHC (Ventanna) and FISH detections of cell block verified *ALK* rearrangement.

We screened 364 patients with advanced NSCLC for *ALK* rearrangements. Twenty-four patients had confirmed *ALK* rearrangements by both IHC and FISH based on their tissue biopsies. Figure 1 shows the representative CT scan, IHC and FISH results for one patient P1. The clinical and demographic characteristics of enrolled patients are summarized in Table 1. Most of the patients are stage IV, with pleura being the most frequent metastatic site. At the time of *ALK* rearrangements detection, 20 of the 24 patients were treatment naïve, while one patient was diagnosed as pleural effusion after adjuvant chemotherapy and another patient progressed from last line of chemotherapy. Blood samples were collected longitudinally from 7 of the 24 patients during their subsequent treatment after the detection of *ALK* rearrangements. Four patients were treated with pemetrexed in combination with cisplatin or carboplatin. One patient P4 was initially treated with pemetrexed and cisplatin and switched to crizotinib after disease progression. Blood samples of these 4 patients were collected prior to the 2nd and 3^rd^ cycle of chemotherapy (Patient 4 had a cycle delay for cycle 3, so we had an additional 4^th^ sample collection at PD after chemotherapy. He was subsequently switched to Crizotinib and his blood sample was also collected at PD after developing resistance against Crizotinib). For the 3 patients only treated with Crizotinib, blood samples were collected after 2 months of treatment. We were able to follow Patient 1 for a longer period of time and collect additional blood samples at PR (4 months of treatment) and at PD (10 months of treatment). (Supplementary Table S1).

**Table 1:** Patient characteristics

Age, year	48.1±10.7
Range	30–66
Gender	
Male	10 (41.7)
Female	14 (58.3)
Stage	
IIIB	2 (8.3)
IV	22 (91.7)
Metastatic site	
Lung	9 (37.5)
Pleura	12 (50)
Bone	4 (16.7)
Brain	6 (25)
Other organs	6 (25)
Multiple organs	14 (58.3)
ECOG score	
0	10 (41.7)
1	13 (54.2)
2	1 (4.2)
Smoking history, pack·yr	
0	14 (58.3)
< 20	7 (29.2)
≥ 20	3 (12.5)
Biopsy origin	
Lung	18 (75)
Pleura	4 (16.7)
Cell block	2 (8.3)
Baseline	
Treatment naïve	20 (83.3)
Disease progression	4 (16.7)
Treatment	
Crizotinib	9 (37.5)
Chemotherapy	10 (41.7)
Unknown	5 (20.8)
No. of blood samples	
1	17 (70.8)
2	3 (12.5)
3	2 (8.3)
≥ 4	2 (8.3)

Note: ECOG: Eastern Cooperative Oncology Group.

### Quality assessment of sequencing data

We performed targeted ultra-deep sequencing on 73 plasma cfDNA samples obtained from 40 patients 24 patients and 36 negative controls with a mean coverage depth of 22,708×. Among all samples, the mapped reads percentage was over 99%. On average, 59.3% of all reads are mapped to our targeted region, demonstrating high capture efficiency of the designed probes. The imputed sizes of all cfDNA fragments ranged from 154 bp to 178 bp, consistent with previous knowledge about the cfDNA length.

### ALK rearrangement detection

**Figure 2:**
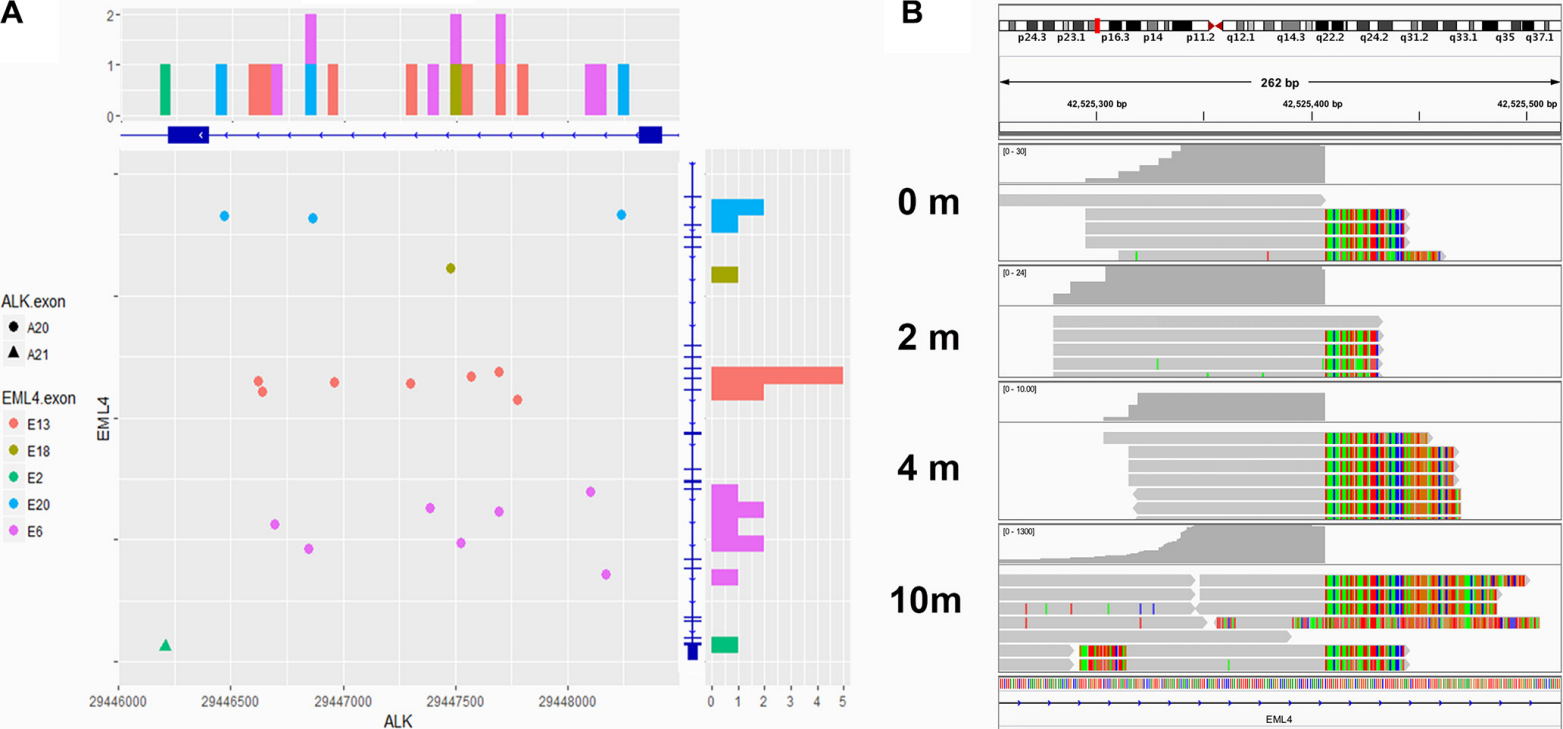
Breakpoints of *EML4-ALK* fusion (**A**) Breakpoints of *EML4-ALK* fusion detected in 19 patients were shown by chromosome coordinates (X axis ~ *ALK*, Y axis ~ *EML4*). Eighteen breakpoints located in the intron 19 of *ALK*, while the other one located in the intron 20. On the EML4 side, the breakpoints were dispersive. Intron 6, 13 and 20 were the regions with most frequent break events. (**B**) The Integrative Genomics Viewer (IGV) diagram shows that the breakpoints on *ALK* were identical among different blood samples from the same patient (P1).

*ALK* rearrangement was detected from 19 of the 24 patients at baseline prior to any treatment, demonstrating a sensitivity of 79.2% (95% CI: 57.9%, 92.9%). We randomly selected 36 *ALK* rearrangements negative cases assessed by FISH and performed NGS. NGS did not detect *ALK* rearrangements in any patients. Therefore, the specificity is 100% (36/36). (Table 2) *ALK* rearrangement presence dropped to 69.2% (9/13) among samples collected after Crizotinib and/or chemotherapy treatment. Furthermore, our results showed the exact breakpoint position on DNA where the rearrangement occurred. We have therefore summarized the breakpoint positions on both *ALK* and *EML4* in all 14 patients with rearrangements detected in Figure 2A. Except for one patient (P4), who has *EML4* fused to *ALK* exon 21, all other patients have *EML4* fused to *ALK* exon 20, with breakpoints on *ALK* residing in intron 19, echoing with previous reports. On the other hand, the breakpoints on *EML4* range across multiple introns, with “hot clusters” in intron 6, 13, and 20. Detailed breakpoint information is listed in Supplementary Table S2. As expected, *ALK* rearrangement breakpoints were always found to be identical among different plasma samples obtained from the same patient. An Integrative Genomics Viewer (IGV) diagram was shown to demonstrate the breakpoints between different blood samples from the same patient (P1) (Figure 2B).

**Table 2:** Performance of *ALK* detection in plasma by NGS

Patients (*n*)^*^		NGS		Total	
		+	–		
FISH	+	19	5	24	Sensitivity 79.2%
	**–**	0	36	36	Specificity 100%
Total		19	41	60	
		PPV 100%	NPV 87.8%		Matching Rate 91.7%

* Only baseline samples were used

### Detection of other mutations

**Figure 3:**
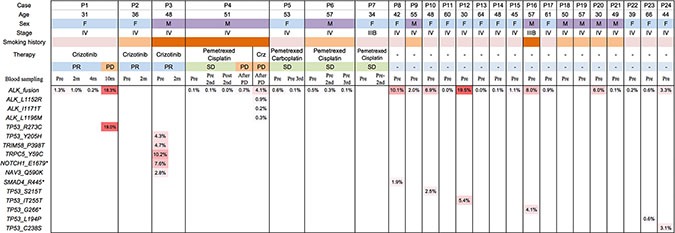
Mutation spectrum In addition to *ALK* rearrangements detected from 19 of 24 patients, mutations in multiple genes including *NAV3*, *NOTCH1*, *TRPC5*, and *TRIM58* were detected in baseline blood sample of Patient 3, and a loss-of-function nonsense mutation in *SMAD4* was shown in Patient 8. Mutations in *TP53* were found in blood sample of 5 patients (P3, P7, P10, P12 and P16).

In addition to *ALK* rearrangement, mutations among all targeted regions were also assessed. As expected, we found that the mutational load of these *ALK* positive patients is generally low, with very few mutations identified besides *ALK* rearrangements. Figure 3 summarizes all mutations detected and their associated AF in all samples. Six of the 24 patients had mutations in other targeted regions at baseline, most of which within *TP53*. Patient 3 carries mutations in multiple genes including *NAV3*, *NOTCH1*, *TRPC5*, and *TRIM58*; in addition, the baseline plasma sample of Patient 8 harbors a *SMAD4* loss-of-function nonsense mutation (Supplementary Table S3).

### ctDNA concentration and tumor dynamics

We next imputed the ctDNA concentration by multiplying the cfDNA concentration with the maximum allele frequency (AF) of all variants detected in each sample. If no variant was detected in a sample, we assume there is no ctDNA present. The ctDNA concentration for each sample is listed in Supplementary Table S4. The imputed ctDNA concentration varies greatly among patients and samples, ranging from 0.66 pg mL^−1^ to more than 1 ng mL^−1^.

**Figure 4:**
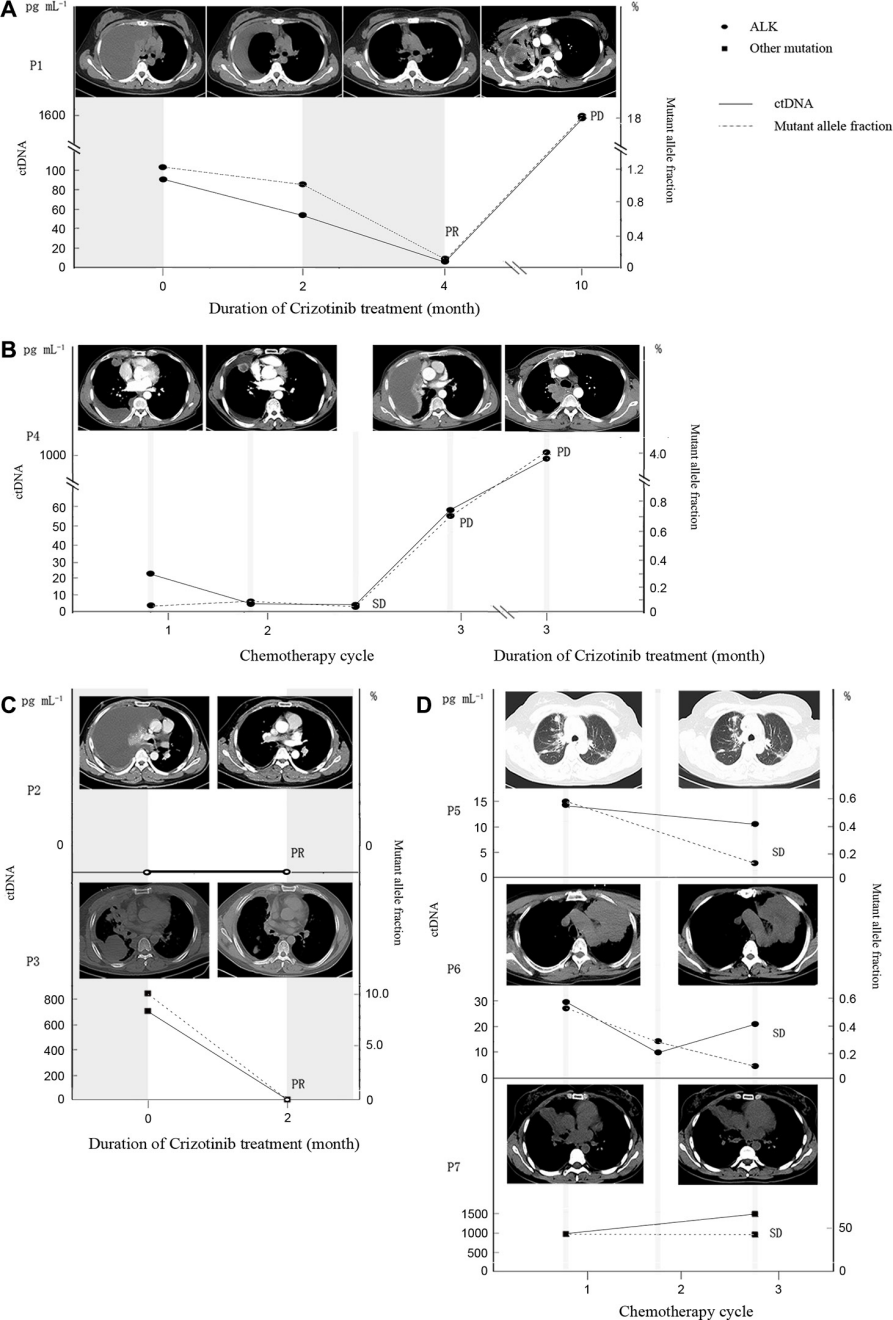
The association among mutant allele fraction of ALK, ctDNA concentration and clinical relevance (**A**–**C**) The association among mutant allele fraction of *ALK*, ctDNA concentration and clinical relevance in patients exposed to Crizotinib (P1, P4 and P2 and P3). P1 and P3 experienced a drop in both mutant allele fraction and ctDNA concentration at PR. P1 and P4 experienced an increase in both mutant allele fraction and ctDNA concentration at PD. P2 had undetectable ctDNA concentration. (**D**) The association among mutant allele fraction, ctDNA concentration and clinical relevance in patients received chemotherapy (P5, P6 and P7). All of them achieved SD and had relatively stable ctDNA concentration and mutant allele fraction.

Among the 7 patients with longitudinal plasma cfDNA, the imputed concentration of ctDNA is largely associated with the disease status assessed by radiological imaging (CT) methodology. Patient 1 achieved PR after Crizotinib treatment and showed a clear drop of ctDNA concentration in plasma after treatment compared to the baseline. She later experienced relapse after post-surgery adjuvant chemotherapy. The main manifestation was pleural effusion with no measurable disease lesion. Both surgical resected samples and cell block from pleural effusion were tested positive for *ALK* rearrangement by both IHC and FISH (Figure 1). The imputed ctDNA concentration decreased from 90.52 pg mL^−1^ to 54.56 pg mL^−1^ after 2 months of Crizotinib treatment, when CT indicated less pleural effusion. Furthermore, after 4 months of Crizotinib treatment, the ctDNA concentration decreased further, declining to 5.43 pg mL^−1^, accompanied with a significant reduction in the allele frequency of *ALK* rearrangements, reaching 0.145%. In the meanwhile, her CT scan revealed an obvious remission of pleural effusion. At PD, after 10 months of treatment, her ctDNA concentration significantly increased to 1601.3 pg mL^−1^, accompanied with an increase in AF of *ALK*, reaching 18.3%. However, no ALK inhibitor resistance mutations were identified. (Figure 4A)

Patient 4 received pemetrexed in combination with cisplatin for 3 cycles before he switched to Crizotinib due to progression. He initially responded to Crizotinib, and achieved PR, but ultimately developed resistance within just 3 months, resulting in disease progressed. His blood samples were collected at baseline, after chemotherapy cycle1, 2, before cycle 3 and at PD after administrating Crizotinib. Change of the imputed ctDNA concentration, along with the allele frequencies of *ALK* arrangements can be found in Figure 4B. The ctDNA concentrations after chemotherapy cycle 1 and 2 were 4.63 pg mL^−1^ and 3.37 pg mL^−1^ respectively, which are significantly lower than the baseline concentration (22.47 pg mL^−1^), accompanied by a reduction in the density within the lesion, suggesting colliquative necrosis within the tumor. However, primary tumor shrinkage was not evident on follow-up CT images, so the efficacy was assessed as stable disease (SD). Due to a one-month delay of cycle 3, another test of plasma cfDNA was conducted at a later time and the ctDNA concentration increased to 58.98 pg mL^−1^, with *ALK* rearrangements AF rising to 0.714%. CT scan was repeated and pleural effusion was increased significantly; therefore, the tumor assessment was PD. Subsequently, the patient was switched to Crizotinib and achieved PR. However, 3 months after the treatment, his CT scan demonstrated PD accompanied with significant increases in ctDNA concentration, rising to 968.7 pg mL^−1^ and AF for ALK, reaching 4.07%. In addition, this patient developed 3 Crizotinib resistant mutations: *ALK* L1152R, *ALK* I1171T and *ALK* L1196M. (Figure 4B)

Patients 2 and 3 both received Crizotinib and achieved PR at their first post-treatment CT assessments. Patient 2 did not show a detectable level of ctDNA mutations at either baseline or PR. Patient 3 had shown a ctDNA concentration of 711.9 pg mL^−1^ at baseline, which dropped to undetectable at PR, consistent with the disease status assessed by CT (Figure 4C).

Patient 5, 6, and 7 all received pemetrexed in combination with cisplatin or carboplatin. Blood samples were collected at various time points from all three patients, and they were all assessed as SD at their last blood collection point. Patient 5 had a baseline ctDNA concentration of 14.79 pg mL^−1^, while his ctDNA became undetectable after the first cycle of chemotherapy and re-elevated to 11.15 pg mL^−1^ after cycle 2. Patient 6 demonstrated a very similar trend as patient 5, with a modest drop of ctDNA concentration after the first cycle of chemotherapy, followed by a slight rebound after cycle 2. ALK rearrangement was not detected from any plasma samples from Patient 7. But a *TP53* G262S mutation was observed before chemotherapy and after cycle 1. The imputed ctDNA concentration was 1011.08 pg mL^−1^ and 1526.68 pg mL^−1^, respectively (Figure 4D). Collectively, our data demonstrated that capture-based targeted sequencing is capable of detecting and quantifying rearrangements as well as other classes of mutations, thus serving as a powerful tool for disease monitoring.

## DISCUSSION

In this study, we demonstrate that *ALK* rearrangements can be identified with high sensitivity and specificity using capture-based deep sequencing of plasma cfDNA. Among the 24 advanced lung adenocarcinoma patients with known *ALK* rearrangements confirmed by their tissue biopsies, *ALK* rearrangements were successfully detected from plasma samples of 19 patients with 79.2% sensitivity and 100% specificity. NGS assay has been reported to detect fusions with 75% (6/8) sensitivity [16]. *ALK* rearrangements were not detected from the baseline plasma samples of 5 patients. There are multiple factors that affect ctDNA detection sensitivity, including cfDNA sample handling and conversion rate during the library preparation. It should also be noted that probe capture efficiency for rearranged DNA fragments could be lower than for wildtype DNA fragments at the same location, since the probes would only have the *ALK* part of the fragments to match on. The capture efficiency would therefore be biased toward the wildtype fragments, and the bias would become greater as the fragment size becomes smaller. With average length of 170 bps, much shorter than the usual DNA fragments from tissue samples, ctDNA makes fusion fragment capturing especially challenging.

One interesting pattern we found in our study is the intra-correlation of the rearrangement detection between different samples obtained from the same patient. All patients who tested positive for *ALK* rearrangements post-treatment also exhibited *ALK* rearrangements at baseline. Patients who tested negative for *ALK* rearrangements at later time points also lack such mutation at baseline (Fisher's exact test, *p* = 0.029). Such trend indicates that there might be by-patient variation of the likelihood of *ALK* rearrangement detection, possibly due to the variation of the amount and type of ctDNA released in each patient. Given such high sensitivity, liquid biopsy could become a useful surrogate for those patients who experience SD or PD after chemotherapy but when re-biopsy is not feasible.

Besides determining the presence of *ALK* rearrangement, NGS-based approach provides interesting details on the rearrangements detected, including the fusion partner and the precise location of the breakpoints on both genes, providing important information for further molecular subtyping of the patient population. Such information cannot be obtained from either IHC or FISH. It has recently been reported that patients harboring different *ALK* fusion variants respond differently to Crizotinib [19], illustrating the importance of accurately identifying the exact variant type, for which neither IHC nor FISH is incapable of.

Furthermore, our study demonstrates the quantification capability of NGS for ctDNA detection in plasma through strong correlation between imputed ctDNA concentration and CT assessment of the disease status. There are multiple reports on the association between ctDNA concentration and the imaging manifestation of cancer [14, 15, 20]. Evidence shows that liquid biopsy may detect change of tumor burden or predict disease relapse even before the imaging signal appears [15].

In addition, capture-based NGS profiles a large range of genetic regions simultaneously and therefore can also identify mutations-mediated drug resistance, thus facilitating and re-directing personalized therapy. A few studies have reported identifying ALK resistance mutations using targeted NGS [21]. We identified 3 resistant mutations in patient 4 after Crizotinib treatment, indicating the possibility of having multiple sub-clones carrying distinct resistant mutations. This patient might be sensitive to Lorlatinib, a third generation ALK inhibitor, which retains potency against all known ALK mutations [22]. Panel sequencing assays offer molecular evidence to study possible resistance mechanism or responsiveness biomarkers which could be applied clinically to support the treatment decision after disease progression.

Our results demonstrated the feasibility of utilizing capture-based targeted deep sequencing to detect *ALK* rearrangements from plasma in advanced NSCLC patients. This provides an alternative to tissue testing in treatment-naive patients when sufficient tissue samples are not available. This technology also serves as a potential noninvasive approach on monitoring tumor burden and detecting secondary resistant mutations in patients undergoing treatment.

## MATERIALS AND METHODS

### Patients and samples

Three hundred and sixty-four patients from the Department of Respiratory and Critical Care Medicine with stage IIIB or IV NSCLC were screened for *ALK* rearrangements at West China hospital from April, 2015 to April, 2016. Patients must be positive for *ALK* rearrangements by both IHC and FISH based on their tissue biopsies collected either at initial NSCLC diagnosis or at the time of disease progression. Twenty-four patients were eligible and enrolled in this study.

Peripheral blood and tissue samples were collected from all patients. Additional blood samples were collected from some patients undergoing either Crizotinib treatment (at month 2, 4, or depending on patients' visit schedule) or chemotherapy (after the 1^st^ or 2^nd^ cycle, or depending on patients' visit schedule). The prospective study was approved by IRB Committee of Sichuan University, and the informed consent was obtained from each patient.

### Ventana ALK IHC Assay

The ALK IHC assay included a highly sensitive anti-ALK (D5F3) rabbit monoclonal primary antibody (Ventana Medical Systems Inc), combined with an OptiView Amplification Kit and an OptiView DAB IHC Detection Kit (Ventana Medical Systems Inc). The automatic staining procedure was performed on a Bench- Mark XT slide stainer (Ventana Medical Systems Inc) according to Ventana ALK IHC Assay's instructions. Simultaneously, each case was stained with a matched rabbit monoclonal negative control immunoglobulin G antibody.

### FISH detection

*ALK*-FISH detection was performed using an LSI ALK Dual Color Break Apart Rearrangement Probe (Abbott-Vysis, Downers Grove, Ill) according to the manufacturer's instructions. Two patterns of *ALK* rearrangement FISH signals have been demonstrated in lung adenocarcinoma: the isolated red signal (IRS) and classic break-apart (BA) patterns. In the BA pattern, the distance between the red and green signal borders should be at least twice the diameter of the larger of the 2 signals. At least 100 nonoverlapping tumor cell nuclei should be counted for interpretation of each case. Positive result was defined as > 15% of tumor cells showing a BA and/or IRS pattern.

### Preparation of plasma cell-free DNA

Ten ml of blood sample was collected in K3EDTA-containing tubes (Cell-Free DNA BCT) and centrifuged at 2000 g for 10 minutes at +4°C within 72 hours after collection. Carefully aspirated plasma supernatant was transferred into fresh 15 ml centrifuge tubes without disturbing the buffy coat layer. The plasma samples were then centrifuged for 10 min at 16,000 g at +4°C and the supernatant was then removed to a new tube with a pipette without disturbing the pellet. The plasma was stored at −80°C until further analysis. Circulating cell-free DNA was recovered from 4 to 5 ml of plasma samples using the QIAamp Circulating Nucleic Acid kit (Qiagen).

### Quantification of plasma cell-free DNA

Quantification of cfDNA was performed using the Qubit 2.0 Fluorometer with the dsDNA HS assay kits (Life Technologies, Carlsbad, CA). The starting material consists of 50 ng cfDNA prepared from Qiagen method for the subsequent testing.

### Capture-based targeted DNA sequencing

DNA was profiled using a commercial available capture-based targeted sequencing panel (Burning Rock Biotech Ltd, Guangzhou China.), targeting 168 genes and spanning 160 K of Human genomic regions. (Supplementary Table S5) DNA was hybridized with the capture probes baits, selected with magnetic beads, and PCR amplified. A bioanalyzer high sensitivity DNA assay was then used to assess the quality and size range. 30 indexed samples were then sequenced on a NextSeq 500 (Illumina, Inc., USA) with pair-end reads.

### Sequence data analysis

Sequence data were mapped to the human genome (hg19) using BWA aligner 0.7.10. Local alignment optimization, variant calling and annotation was performed using GATK 3.2, MuTect, and VarScan. DNA translocation analysis was performed using both Tophat2 and Factera 1.4.3.

## SUPPLEMENTARY MATERIALS TABLES








