# Supplementary material for: Noninvasive genotyping and monitoring of anaplastic lymphoma kinase (ALK) rearranged non-small cell lung cancer by capture-based next-generation sequencing
Source: Oncotarget. 2016 Aug 24;7(40):65208–17. doi: 10.18632/oncotarget.11569 (PMC5323149; doi:10.18632/oncotarget.11569)
Supplement: Supplementary file 1 [file oncotarget-07-65208-s001.pdf]

Noninvasive genotyping and monitoring of anaplastic lymphoma kinase (ALK) rearranged non-small cell lung cancer by capture-based next-generation sequencing

Supplementary Materials

Supplementary Table S1: Summary of the next generation sequencing data.
See Supplementary_ Table_S1

Supplementary Table S2: ALK fusions detected in 19 patients

Patient ID	Gene	Baseline AF	EML4_break Point	ALK_break Point	EML4:exon	ALK:exon	HGVs format
P01	ALK	1.26%	42525721	29447302	E13	A20	EML4:p.Met1_Lys496;ALK:p.Val1058_*1621
P04	ALK	0.05%	42482380	29446207	E2	A21	EML4:p.Met1_Lys69;ALK:p.Gly1121_*1621
P05	ALK	0.59%	42504599	29447692	E6	A20	EML4:p.Met1_Gln222;ALK:p.Val1058_*1621
P06	ALK	0.50%	42507968	29448100	E6	A20	EML4:p.Met1_Gln222;ALK:p.Val1058_*1621
P08	ALK	10.09%	42525792	29446961	E13	A20	EML4:p.Met1_Lys496;ALK:p.Val1058_*1621
P09	ALK	1.98%	42544475	29447478	E18	A20	EML4:p.Met1_Ile685;ALK:p.Val1058_*1621
P10	ALK	6.85%	42499463	29447524	E6	A20	EML4:p.Met1_Gln222;ALK:p.Val1058_*1621
P11	ALK	0.02%	42494395	29448171	E6	A20	EML4:p.Met1_Gln222;ALK:p.Val1058_*1621
P12	ALK	19.47%	42524339	29446641	E13	A20	EML4:p.Met1_Lys496;ALK:p.Val1058_*1621
P13	ALK	0.03%	42553276	29448240	E20	A20	EML4:p.Met1_Tyr747;ALK:p.Leu1058_*1621
P14	ALK	0.09%	42552770	29446866	E20	A20	EML4:p.Met1_Tyr747;ALK:p.Leu1058_*1621
P15	ALK	1.05%	42553182	29446472	E20	A20	EML4:p.Met1_Tyr747;ALK:p.Leu1058_*1621
P16	ALK	8.01%	42505286	29447385	E6	A20	EML4:p.Met1_Gln222;ALK:p.Val1058_*1621
P17	ALK	0.91%	42527554	29447695	E13	A20	EML4:p.Met1_Lys496;ALK:p.Val1058_*1621
P20	ALK	6.04%	42526035	29446620	E13	A20	EML4:p.Met1_Lys496;ALK:p.Val1058_*1621
P21	ALK	0.11%	42523040	29447778	E13	A20	EML4:p.Met1_Lys496;ALK:p.Val1058_*1621
P22	ALK	0.16%	42498570	29446844	E6	A20	EML4:p.Met1_Gln222;ALK:p.Val1058_*1621
P23	ALK	0.63%	42502563	29446697	E6	A20	EML4:p.Met1_Gln222;ALK:p.Val1058_*1621
P24	ALK	3.34%	42526715	29447568	E13	A20	EML4:p.Met1_Lys496;ALK:p.Val1058_*1621

Supplementary Table S3: Putative somatic mutations detected in patients ctDNA

patient ID	Sample ID	Gene	mutation type	Exon number	Amino acid change	Allele fraction	chr	pos	Ref	Alt	dbSNP_ID	COSMIC_ID
P01	RS1600890PLA	TP53	missense_variant	8	R273C	18.95%	17	7577121	G	A	rs121913343	COSM1645518
P03	RS1501596PLA	TRIM58	missense_variant	6	P398T	4.73%	1	248039522	C	A	NA	COSM355355
P03	RS1501596PLA	TP53	missense_variant	6	Y205H	4.27%	17	7578236	A	G	NA	COSM43642
P03	RS1501596PLA	TRPC5	missense_variant	2	Y59C	10.17%	X	111195473	T	C	NA	NA
P03	RS1501596PLA	NOTCH1	stop_gained	27	E1679*	7.56%	9	139397766	C	A	NA	COSM3724366
P03	RS1501596PLA	NAV3	missense_variant	8	Q590K	2.76%	12	78401086	C	A	NA	NA
P04	RS1603970PLA	ALK	missense_variant	22	L1152R	0.91%	2	29445270	A	C	NA	COSM97185
P04	RS1603970PLA	ALK	missense_variant	23	L1196M	0.34%	2	29443631	G	T	NA	COSM99137
P04	RS1603970PLA	ALK	missense_variant	22	I1171T	0.15%	2	29445213	A	G	NA	COSM4381100
P08	RS1501823PLA	SMAD4	stop_gained	11	R445*	1.93%	18	48603032	C	T	rs377767360	COSM14096
P10	RS1500734PLA	TP53	missense_variant	6	S215T	2.50%	17	7578205	C	G	NA	COSM44175
P12	RS1500572PLA	TP53	disruptive_ inframe_deletion	7	IT255T	5.44%	17	7577514	GTGA	G	NA	COSM1480063
P16	RS1501592PLA	TP53	stop_gained	8	G266*	4.09%	17	7577142	C	A	NA	COSM44891
P23	RS1604133PLA	TP53	missense_variant	6	L194P	0.59%	17	7578268	A	G	N	COSM3675531
P24	RS1501547PLA	TP53	missense_variant	7	C238S	3.08%	17	7577569	A	T	N	COSM4340290

Supplementary Table S4: Consentrations and Allelic Frequencies of ctDNA.
See Supplementary_ Table_S4

Supplementary Table S5: Genomic regions targeted by the capture panel.
See Supplementary_ Table_S5
