# Supplementary material for: Noninvasive genotyping and monitoring of anaplastic lymphoma kinase (ALK) rearranged non-small cell lung cancer by capture-based next-generation sequencing
Source: Oncotarget. 2016 Aug 24;7(40):65208–17. doi: 10.18632/oncotarget.11569 (PMC5323149; doi:10.18632/oncotarget.11569)
Supplement: Supplementary file 2 [file oncotarget-07-65208-s002.docx]

| **Supplementary Table 1 Summary of the next generation sequencing data** | | | | | | | |  |  |  |
| --- | --- | --- | --- | --- | --- | --- | --- | --- | --- | --- |
| Sample.ID | total reads | mapped reads | insert Size | covered region reads | rmdup.covered region.reads | library complexity | mean depth | median depth | percent.depth less.than.1000 | target ratio |
| RS1500425PLA | 145104994 | 144589984 | 173 | 84934958 | 11201961 | 13.2% | 29630 | 29436 | 0.2% | 58.53% |
| RS1500426PLA | 151465981 | 150815750 | 170 | 91560715 | 10017251 | 10.9% | 32044 | 32242 | 0.2% | 60.45% |
| RS1500572PLA | 134374304 | 133848981 | 170 | 77256533 | 8490061 | 11.0% | 26702 | 26165 | 0.2% | 57.49% |
| RS1500607PLA | 168745451 | 168080338 | 172 | 99662525 | 8534488 | 8.6% | 34854 | 34326 | 0.2% | 59.06% |
| RS1500656PLA | 182146044 | 181092124 | 170 | 108696502 | 14177329 | 13.0% | 37892 | 37897 | 0.2% | 59.68% |
| RS1500726PLA | 168276027 | 167549168 | 165 | 105776721 | 13247993 | 12.5% | 36639 | 35959 | 0.2% | 62.86% |
| RS1500734PLA | 143810668 | 142572116 | 172 | 86474451 | 11894953 | 13.8% | 30370 | 30669 | 0.2% | 60.13% |
| RS1500798PLA | 61801441 | 61595432 | 173 | 33723122 | 3224103 | 9.6% | 25782 | 26142 | 1.6% | 54.57% |
| RS1500827PLA | 75904682 | 75614309 | 169 | 41487437 | 4716509 | 11.4% | 31404 | 32099 | 1.4% | 54.66% |
| RS1500930PLA | 44997694 | 44822928 | 165 | 6940751 | 3099012 | 44.6% | 5348 | 5524 | 3.5% | 15.42% |
| RS1500955PLA | 99674564 | 99383869 | 166 | 54925607 | 5644497 | 10.3% | 41847 | 42117 | 1.4% | 55.10% |
| RS1500988PLA | 41923175 | 41811921 | 172 | 23322214 | 2358721 | 10.1% | 17777 | 18084 | 1.6% | 55.63% |
| RS1501024PLA | 42825564 | 42714467 | 170 | 23657963 | 2491609 | 10.5% | 18036 | 18337 | 1.6% | 55.24% |
| RS1501119PLA | 46810362 | 46616401 | 163 | 27070134 | 5906227 | 21.8% | 20825 | 21201 | 0.6% | 57.83% |
| RS1501121PLA | 39246932 | 39073216 | 168 | 20279993 | 2353986 | 11.6% | 15676 | 16301 | 0.8% | 51.67% |
| RS1501122PLA | 42283610 | 42113262 | 169 | 29386564 | 4772039 | 16.2% | 22568 | 23069 | 0.6% | 69.50% |
| RS1501161PLA | 44528223 | 44351336 | 169 | 30673697 | 4967315 | 16.2% | 23615 | 24055 | 0.4% | 68.89% |
| RS1501310PLA | 46933898 | 46734385 | 167 | 32029816 | 4822127 | 15.1% | 24629 | 25227 | 0.4% | 68.24% |
| RS1501547PLA | 65099122 | 64837395 | 167 | 42092890 | 4283469 | 10.2% | 32283 | 32856 | 0.4% | 64.66% |
| RS1501550PLA | 37850983 | 37701674 | 170 | 24235353 | 3381075 | 14.0% | 18821 | 19655 | 0.6% | 64.03% |
| RS1501592PLA | 36899726 | 36736625 | 167 | 24840511 | 4671061 | 18.8% | 18917 | 19129 | 0.2% | 67.32% |
| RS1501593PLA | 32544645 | 32405423 | 170 | 21891775 | 656377 | 3.0% | 16817 | 17039 | 0.2% | 67.27% |
| RS1501596PLA | 45123219 | 44967002 | 166 | 30817277 | 5334402 | 17.3% | 23564 | 24145 | 0.6% | 68.30% |
| RS1501597PLA | 33984605 | 33852014 | 171 | 22600053 | 1427835 | 6.3% | 17626 | 18124 | 0.8% | 66.50% |
| RS1501660PLA | 44273297 | 44036263 | 169 | 28582198 | 3158658 | 11.1% | 21848 | 22364 | 0.6% | 64.56% |
| RS1501777PLA | 43648659 | 43469920 | 168 | 19545797 | 2210271 | 11.3% | 15167 | 15698 | 1.4% | 44.78% |
| RS1501778PLA | 37906847 | 37765783 | 168 | 24636314 | 3736855 | 15.2% | 19056 | 19621 | 1.2% | 64.99% |
| RS1501823PLA | 43793866 | 43605636 | 168 | 29489678 | 6362783 | 21.6% | 22600 | 22947 | 0.6% | 67.34% |
| RS1502215PLA | 37332319 | 37182353 | 174 | 25002596 | 4574174 | 18.3% | 19292 | 19838 | 0.8% | 66.97% |
| RS1502221PLA | 41158115 | 41009475 | 169 | 27783240 | 6107994 | 22.0% | 21486 | 21872 | 0.2% | 67.50% |
| RS1600890PLA | 59647178 | 59439471 | 166 | 34884137 | 5975839 | 17.1% | 26939 | 27258 | 0.2% | 58.48% |
| RS1601007PLA | 47359072 | 47208457 | 168 | 28767371 | 3691224 | 12.8% | 22470 | 23213 | 0.2% | 60.74% |
| RS1601305PLA | 46107042 | 45971040 | 167 | 28461846 | 5456827 | 19.2% | 22158 | 22938 | 0.2% | 61.73% |
| RS1601808PLA | 41821063 | 41728442 | 168 | 26979554 | 6595084 | 24.4% | 20870 | 21270 | 0.6% | 64.51% |
| RS1601809PLA | 44057025 | 43905904 | 168 | 26388835 | 3826013 | 14.5% | 20415 | 20956 | 0.4% | 59.90% |
| RS1603970PLA | 51665250 | 51569841 | 165 | 28417133 | 5258788 | 18.5% | 21630 | 21846 | 0.8% | 55.00% |
| RS1604133PLA | 52586771 | 52567107 | 166 | 35140212 | 5994489 | 17.1% | 27134 | 27620 | 0.2% | 66.82% |
| RS1500657PLA | 38038512 | 38015321 | 171 | 20517182 | 1122085 | 5.5% | 15494 | 15583 | 1.6% | 53.94% |
| RS1500660PLA | 64965525 | 64919942 | 167 | 32875040 | 2359933 | 7.2% | 25173 | 26040 | 0.6% | 50.60% |
| RS1500808PLA | 49722025 | 49686555 | 175 | 27352953 | 775577 | 2.8% | 20699 | 21065 | 11.4% | 55.01% |
| RS1500809PLA | 47228230 | 47217638 | 172 | 26726547 | 1858994 | 7.0% | 19660 | 19065 | 1.6% | 56.59% |
| RS1500871PLA | 53615940 | 53580575 | 170 | 31389830 | 1601682 | 5.1% | 23743 | 24146 | 1.4% | 58.55% |
| RS1500873PLA | 41110184 | 41100183 | 177 | 23627822 | 1414076 | 6.0% | 17788 | 17956 | 1.6% | 57.47% |
| RS1500903PLA | 37121885 | 37103011 | 169 | 24679292 | 1191273 | 4.8% | 18675 | 19026 | 1.6% | 66.48% |
| RS1500907PLA | 41166624 | 41146766 | 174 | 26843009 | 1630373 | 6.1% | 20389 | 20816 | 1.4% | 65.21% |
| RS1500908PLA | 34444057 | 34425867 | 170 | 22569063 | 1350445 | 6.0% | 17073 | 17353 | 1.6% | 65.52% |
| RS1500912PLA | 42955801 | 42928919 | 173 | 23725130 | 1467706 | 6.2% | 18012 | 18314 | 1.6% | 55.23% |
| RS1500913PLA | 33743198 | 33725349 | 175 | 22095296 | 1236147 | 5.6% | 16790 | 16989 | 1.6% | 65.48% |
| RS1500916PLA | 36713080 | 36689827 | 173 | 22554841 | 1163422 | 5.2% | 17120 | 17420 | 1.6% | 61.44% |
| RS1500925PLA | 39685502 | 39662438 | 154 | 20832066 | 2225263 | 10.7% | 15699 | 14749 | 2.7% | 52.49% |
| RS1500956PLA | 35434974 | 35415403 | 175 | 21837381 | 1044390 | 4.8% | 16664 | 16945 | 1.8% | 61.63% |
| RS1500992PLA | 49986903 | 49924929 | 169 | 8026701 | 1182499 | 14.7% | 6084 | 6192 | 3.5% | 16.06% |
| RS1500994PLA | 54597782 | 54536103 | 169 | 8718737 | 1373870 | 15.8% | 6629 | 6867 | 3.7% | 15.97% |
| RS1501021PLA | 47975255 | 47954047 | 167 | 30735748 | 1916217 | 6.2% | 23279 | 23446 | 1.2% | 64.07% |
| RS1501022PLA | 50800489 | 50779999 | 164 | 32242491 | 2758692 | 8.6% | 24302 | 24081 | 1.2% | 63.47% |
| RS1501037PLA | 48208190 | 48180275 | 170 | 33408444 | 1461666 | 4.4% | 24924 | 25326 | 1.6% | 69.30% |
| RS1501040PLA | 41894727 | 41878315 | 178 | 28479164 | 977114 | 3.4% | 21089 | 21037 | 1.6% | 67.98% |
| RS1501041PLA | 37121587 | 37104160 | 174 | 25370552 | 1476686 | 5.8% | 19074 | 19271 | 1.8% | 68.34% |
| RS1501123PLA | 47090789 | 47054736 | 173 | 24090617 | 2203182 | 9.1% | 18424 | 19113 | 1.0% | 51.16% |
| RS1501140PLA | 44514639 | 44492115 | 171 | 30062071 | 1419922 | 4.7% | 22832 | 23468 | 1.6% | 67.53% |
| RS1501141PLA | 42992151 | 42973602 | 168 | 29120873 | 1284218 | 4.4% | 21991 | 22473 | 1.8% | 67.74% |
| RS1501157PLA | 47607244 | 47580042 | 168 | 33324617 | 1063969 | 3.2% | 24820 | 25170 | 1.4% | 70.00% |
| RS1501337PLA | 53441613 | 53395107 | 171 | 26135719 | 2824685 | 10.8% | 19956 | 20500 | 1.0% | 48.91% |
| RS1501338PLA | 47240072 | 47199592 | 174 | 23096027 | 1415315 | 6.1% | 17685 | 18110 | 0.8% | 48.89% |
| RS1501339PLA | 61007936 | 60975002 | 170 | 36449556 | 2665992 | 7.3% | 27819 | 28180 | 0.6% | 59.75% |
| RS1501340PLA | 54262213 | 54232236 | 170 | 33756461 | 2004239 | 5.9% | 25803 | 26415 | 0.6% | 62.21% |
| RS1501656PLA | 63478885 | 63437187 | 172 | 37900371 | 2915405 | 7.7% | 29070 | 29748 | 0.4% | 59.71% |
| RS1502333PLA | 61781710 | 61750589 | 169 | 40246694 | 2849173 | 7.1% | 30724 | 31143 | 0.4% | 65.14% |
| RS1502338PLA | 62848014 | 62813563 | 167 | 39895577 | 3114281 | 7.8% | 30269 | 30583 | 0.4% | 63.48% |
| RS1502435PLA | 47282107 | 47227082 | 172 | 30306906 | 1568135 | 5.2% | 23154 | 23777 | 0.6% | 64.10% |
| RS1502634PLA | 70244970 | 70173313 | 167 | 42266425 | 3403160 | 8.1% | 32349 | 33150 | 0.4% | 60.17% |
| RS1502721PLA | 64643045 | 64562889 | 169 | 39188456 | 3200059 | 8.2% | 29912 | 30843 | 0.4% | 60.62% |
| RS1503691PLA | 65992981 | 65917332 | 168 | 39499226 | 3375532 | 8.5% | 29826 | 30259 | 0.6% | 59.85% |
