# Supplementary material for: Noninvasive genotyping and monitoring of anaplastic lymphoma kinase (ALK) rearranged non-small cell lung cancer by capture-based next-generation sequencing
Source: Oncotarget. 2016 Aug 24;7(40):65208–17. doi: 10.18632/oncotarget.11569 (PMC5323149; doi:10.18632/oncotarget.11569)
Supplement: Supplementary file 3 [file oncotarget-07-65208-s003.docx]

| **Supplementary Table 4 Consentrations and Allelic Frequencies of ctDNA** | | | | | | | | |  | | |  | |  | |  | |  | |
| --- | --- | --- | --- | --- | --- | --- | --- | --- | --- | --- | --- | --- | --- | --- | --- | --- | --- | --- | --- |
| Case | Age | Sex | Stage | TNM | Smoking history | Biopsy origin | Therapy | | | | Blood sampling | | ALK | | | | | |  |
|  |  |  |  |  |  |  |  |  |  |  |  |  | Fusion | | ctDNA (%) | | ctDNA (pg/mL) | |  |
| P1 | 31 | F | IV | TxNxM1b | None | Pleural effusion  cell block | Crizotinib | PR | | Pre-treatment | | | E13;A20 | | 1.26 | | 90.5 | |  |
|  |  |  |  |  |  |  |  |  |  | 2 month | | |  |  | 1.04 | | 54.9 | |  |
|  |  |  |  |  |  |  |  |  |  | 4 month | | |  |  | 0.15 | | 5.4 | |  |
|  |  |  |  |  |  |  |  | PD | | 10 month | | |  |  | 18.30 | | 1601.3 | |  |
| P2 | 36 | F | IV | T4N2M1a | Light | Lung(PT) | Crizotinib | PR | | Pre-treatment | | | - | | - | | 0.0 | |  |
|  |  |  |  |  |  |  |  |  |  | 2 month | | |  |  | - | | 0.0 | |  |
| P3 | 48 | M | IV | T4N2M1a | Heavy | Lung(PT) | Crizotinib | PR | | Pre-treatment | | | - | | - | | 711.9 | |  |
|  |  |  |  |  |  |  |  |  |  | 2 month | | |  |  | - | | 0.0 | |  |
| P4 | 51 | M | IV | T3N2M1b | Heavy | Lung(PT) | Pemetrexed Cisplatin | SD | | Pre-treatment | | | E2;A21 | | 0.05 | | 22.5 | |  |
|  |  |  |  |  |  |  |  |  |  | Pre-2nd cycle | | |  |  | 0.11 | | 4.6 | |  |
|  |  |  |  |  |  |  |  |  |  | Post-2nd cycle | | |  |  | 0.03 | | 3.4 | |  |
|  |  |  |  |  |  |  |  | PD | | **Pre-3rd cycle*** | | |  |  | 0.71 | | 59.0 | |  |
|  |  |  |  |  |  |  | Crizotinib | PD | | After PD | | |  |  | 4.07 | | 968.7 | |  |
| P5 | 53 | F | IV | T4N2M1b | None | Lung(MS) | Pemetrexed Carboplatin | SD | | Pre-treatment | | | E6;A20 | | 0.59 | | 14.8 | |  |
|  |  |  |  |  |  |  |  |  |  | Pre-2nd cycle | | |  |  | - | | - | |  |
|  |  |  |  |  |  |  |  |  |  | Pre-3rd cycle | | |  |  | 0.13 | | 11.1 | |  |
| P6 | 57 | M | IV | T4N2M1b | Light | Lung(PT) | Pemetrexed Cisplatin | SD | | Pre-treatment | | | E6;A20 | | 0.50 | | 29.5 | |  |
|  |  |  |  |  |  |  |  |  |  | Pre-2nd cycle | | |  |  | 0.29 | | 10.3 | |  |
|  |  |  |  |  |  |  |  |  |  | Pre-3rd cycle | | |  |  | 0.12 | | 22.3 | |  |
| P7 | 34 | F | IIIB | T4N3M0 | None | Lung(PT) | Pemetrexed Cisplatin | SD | | Pre-treatment | | | - | | - | | 1011.1 | |  |
|  |  |  |  |  |  |  |  |  |  | Pre-2nd cycle | | |  |  | - | | 1526.3 | |  |
| P8 | 42 | F | IV | T4N2M1a | None | Pleura(MS) | - | - | | Pre-treatment | | | E13;A20 | | 10.09 | | 518.4 | |  |
| P9 | 55 | M | IV | T4N2M1b | Light | Lung(PT) | - | - | | Pre-treatment | | | E18;A20 | | 1.98 | | 102.9 | |  |
| P10 | 48 | F | IV | T3N3M1b | None | Pleura(MS) | - | - | | Pre-treatment | | | E6;A20 | | 6.85 | | 462.2 | |  |
| P11 | 60 | F | IV | T3N2M1a | None | Pleura(MS) | - | - | | Pre-treatment | | | E6;A20 | | 0.02 | | 2.7 | |  |
| P12 | 30 | F | IV | T2N3M1a | None | Lung(PT) | - | - | | Pre-treatment | | | E13;A20 | | 19.47 | | 629.8 | |  |
| P13 | 64 | F | IV | T4N1M1a | None | Pleura(MS) | - | - | | Pre-treatment | | | E20;A20 | | 0.03 | | 0.7 | |  |
| P14 | 48 | F | IV | T3N3M1b | None | Lung(PT) | - | - | | Pre-treatment | | | E20;A20 | | 0.09 | | 3.9 | |  |
| P15 | 45 | F | IV | T2N2M1b | None | Lung(PT) | - | - | | Pre-treatment | | | E20;A20 | | 1.05 | | 76.2 | |  |
| P16 | 57 | M | IIIB | T3N3M0 | Heavy | Lung(PT) | - | - | | Pre-treatment | | | E6;A20 | | 8.01 | | 785.3 | |  |
| P17 | 61 | F | IV | T4N2M1b | None | Lung(PT) | - | - | | Pre-treatment | | | E13;A20 | | 0.91 | | 50.4 | |  |
| P18 | 50 | M | IV | T3N3M1b | Light | Lung(PT) | - | - | | Pre-treatment | | | - | | - | | 0.0 | |  |
| P19 | 57 | M | IV | T4N1M1a | Light | Lung(PT) | - | - | | Pre-treatment | | | - | | - | | 0.0 | |  |
| P20 | 30 | M | IV | T2N3M1b | Light | Pleural effusion Cell block | - | - | | Pre-treatment | | | E13;A20 | | 6.04 | | 1183.8 | |  |
| P21 | 49 | M | IV | T2N3M1b | Light | Lung(PT) | - | - | | Pre-treatment | | | E13;A20 | | 0.11 | | 11.2 | |  |
| P22 | 39 | F | IV | T1N3M1a | None | Lung(PT) | - | - | | Pre-treatment | | | E6;A20 | | 0.16 | | 7.4 | |  |
| P23 | 66 | F | IV | T4NN3M1b | None | Lung(MS) | - | - | | Pre-treatment | | | E6;A20 | | 0.63 | | 147.5 | |  |
| P24 | 44 | F | IV | T2N3M1b | None | Lung(MS) | - | - | | Pre-treatment | | | E13;A20 | | 3.34 | | 282.9 | |  |
